# Bacterial Community Composition and Function of Tropical River Ecosystem along the Nandu River on Hainan Island, China

**DOI:** 10.3390/ijerph20010382

**Published:** 2022-12-26

**Authors:** Jinbiao Li, Yangni Zhai, Guojian Ge, Yang Xu, Can Wang, Anyong Hu, Yujie Han, Nan Shan, Bo Liu, Jinlin Chen, Wenlin Wang

**Affiliations:** 1 School of Geographic Science, Nantong University, Nantong 226019, China; 2 Nanjing Institute of Environmental Sciences, Ministry of Environmental Protection, Nanjing 210042, China; 3 State Environmental Protection Scientific Observation and Research Station for Ecological Environment of Lake Hulun Wetland, Hulunbuir 021000, China; 4 Co-Innovation Center for Sustainable Forestry in Southern China, Nanjing Forestry University, Nanjing 210037, China

**Keywords:** tropical river, high-throughput sequencing, bacterial diversity, function prediction

## Abstract

Microorganisms play a pivotal role in nutrient cycling in aquatic ecosystems. Understanding bacterial diversity and its functional composition are crucial for aquatic ecology and protection. We investigated the bacterial community structure using 16S rRNA gene amplicons high-throughput sequencing in this study. Results showed that 105 amplicon sequence variants (ASVs) account for 43.8% of the total sequences shared by the Nandu River’s lower, middle, and upper reach and the Songtao Reservoir. The dominant bacterial phylum in the Nandu River and its connected Songtao Reservoir were Proteobacteria and Actinobacteriota, respectively. The highest Chao1 and Shannon index values were found in the lower reach of the Nandu River. Beta diversity analysis showed the divergence in bacterial communities in the Nandu River and Songtao Reservoir, but not in different reaches. Among the water properties, based on the Mantel test, dissolved oxygen, total nitrogen, and nitrite significantly affected the bacterial communities. The functional profiles predicted by Tax4Fun2 showed that metabolism was the most abundant function group. The relative abundance of genetic information processing was significantly higher in the Songtao Reservoir than in the Nandu River, while the relative abundance of human diseases was significantly lower in the Songtao Reservoir than in the Nandu River. The appearance of the xenobiotics biodegradation and metabolism function group requires us to pay more attention to possible water pollution, especially at the upper reach of the Nandu River.

## 1. Introduction

Rivers link land and sea and play an essential role in ecosystems’ material circulation and energy flow [1]. Pesticides, heavy metals, and agricultural activities easily pollute water bodies, thereby affecting their ecological function [2,3,4]. On the other hand, water sources such as rivers may be subject to microbial contamination from human or animal feces and wastewater discharge [5,6,7]. At the same time, in aquatic ecosystems, microbial activity is of great importance to ecosystem function [8]. As decomposers and mineralizers, bacteria can decompose organic pollutants into inorganic chemical components and purify water, eventually affecting and regulating the quality of water bodies. Thus, understanding the bacterial diversity and composition of the river system is fundamental to better managing it.

The Nandu River is the largest river on Hainan Island and flows through seven cities and counties. The river is 314 km long and has a discharge of 6099 billion cubic meters, with a population of more than 2 million people living around its drainage area. It is the primary source of living water for Haikou City and other regions. The Nandu River has been polluted to varying degrees due to rapid industrial and economic development, but it has also polluted adjacent coastal waters [9,10,11]. On the other hand, problems such as garbage dumping, sewage disposal, and deforestation are relatively serious at the upper reach of the Nandu River, and this has threatened the water quality. The Songtao Reservoir, located at the upper reach of the Nandu River, is the largest reservoir on Hainan Island, one of the 10 largest reservoirs in China, and it is a significant source of production and domestic water supply for cities such as Danzhou, Lingao, Tunchang, Chengmai, Dingan, Haikou, and Yangpu. Recently, several studies have been conducted evaluating the composition, distribution, and source of heavy metals [12] and nutrients such as nitrogen and phosphorus [10] in the Nandu River. However, no study has assessed the composition and diversity of the bacterial community of the Nandu River.

Many methods have been used to detect and identify microbial in aquatic ecosystems, including culturing, biochemical reactions, and immunological methods [13]. However, most of these methods are difficult and time-consuming [14]. Recently, Next Generation Sequencing (NGS) has provided molecular methods for establishing a baseline for monitoring environmental perturbations. NGS can also help researchers identify possible pathogens in the aquatic systems and discover new water quality bioindicators [15,16].

Factors governing the distribution of bacterial communities vary due to the different locations and environments of the aquatic ecosystems. For instance, Aguilar et al. deemed that climate warming could support a higher diversity of microbial communities based on a study conducted in the Andean plateau lakes [17]. Godoy et al. found that phosphate, ammonia-nitrate, and dissolved oxygen were important factors that structured bacterial communities in a heavily polluted river in Brazil [18]. Moreover, Wang et al. found that besides nutrients, metals could also drive the microbial community composition in urban rivers [19]. However, few studies of the freshwater on Hainan Island, China, have been published to address the bacterial composition and its driving factors. The objectives of this study were to determine: (1) the degree of heterogeneity of bacterial community composition in different reaches of the Nandu River and Songtao Reservoir; (2) the relationship between environmental factors and changes in bacterial composition; (3) the functional diversity of the bacterial communities of the Nandu River and Songtao Reservoir.

## 2. Materials and Methods

### 2.1. Sample Collection

**Figure 1:**
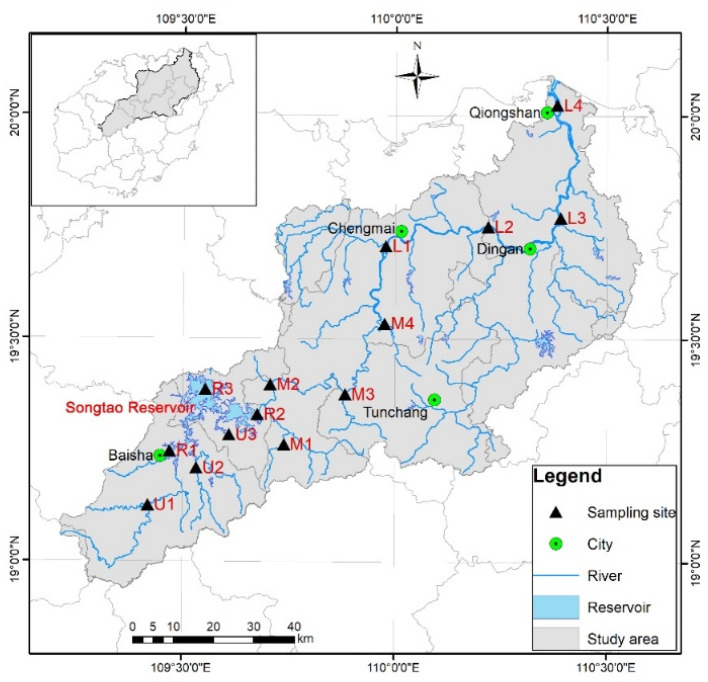
Sampling sites in the Nandu River.

The Nandu River is the largest river on Hainan Island, China, and flows from the center to the north of Hainan Island. This island has a tropical marine monsoon climate, with a mean annual precipitation of approximately 1700 mm, mean annual temperature of 23.8 °C, and abundant water resources [20]. Even though the Nandu River has a relatively short flow, its volume of water is large and flow regions are complex. The upper (U), middle (M), and lower (L) reaches of the Nandu River were divided by the Songtao Reservoir Dam and Jiulong Shoal. Water samples were collected in November 2021 from 14 locations of the Nandu River (U1~U3, M1~M4, and L1~L4) and Songtao Reservoir (R1~R3), respectively (Figure 1). For the sampling points, U1~U3 were located at the upper reach, M1~M4 were located at the middle reach, and L1~L4 were located at the lower reach, while R1~R3 were sampled from the Songtao Reservoir.

In order to avoid the potential impact of temperature changes, all 14 samples were collected between 10 am and 2 pm for three days. When sampling, three liters of water from each sampling point were collected and maintained in sterile plastic bottles. The samples were then stored in a cooling box with dry ice filled in it until the filtering process, which occurred no later than 24 h after sampling. For water chemical properties, samples were filtered through 0.45 μm Millipore GS membranes of cellulose ester (47 mm diameter, white, smooth—©Merck KGaA, Darmstadt, Germany). For DNA extraction, each sample was filtered through 0.22 μm Millipore GS membranes of cellulose ester (47 mm diameter, white, smooth—©Merck KGaA, Darmstadt, Germany) [21].

### 2.2. Water Property

The water quality indexes integrate water temperature, pH, dissolved oxygen (DO), electrical conductivity (EC), turbidity (NTU), total nitrogen (Total N), total phosphorus (Total P), chemical oxygen demand (permanganate index, COD_Mn_), ammoniacal nitrogen (NH_4_^+^), nitrate (NO_3_^−^), nitrite (NO_2_^−^), and phosphate (PO_4_^3−^). Water temperature, pH, EC, and NTU were determined immediately after sampling. Water temperature, pH, EC, and DO were measured using a digital portable multimeter (Multi 3630, WTW^®^, Weilheim, Germany). Water turbidity was measured using a portable turbidimeter (2100Q, HACH^®^, Loveland, CO, USA). Water TN was determined using alkaline potassium persulfate digestion UV spectrophotometric method [22]. Water TP was determined using the ammonium molybdate spectrophotometric method [23]. The permanganate index was determined according to ISO 8467-1993 [24]. The concentrations of NH_4_^+^, NO_3_^−^, NO_2_^−^, and PO_4_^3−^ in the water were analyzed using a continuous flow analyzer (Skala San++, Skalar Analytical B.V., Breda, Netherlands).

### 2.3. DNA Extraction, PCR Amplification, and Sequencing

Total DNA was extracted from the membranes using the Power Soil DNA Isolation Kit^®^ (MoBio Labs, Inc. Solana Beach, CA, USA) according to the manufacturer’s instructions. In order to assess the success of the extraction, the V3-V4 16S region of bacterial ribosomal RNA was amplified using the primers 338F (5′-ACTCCTACGGGAGGCAGCAG-3′) and 806R (5′-GGACTACHVGGGTWTCTAAT-3′) [25], and visualized on 2% agarose gels in TAE buffer (400 mM Tris, 20 mM glacial acetic acid, 1 mM EDTA). The polymerase chain reaction (PCR) amplification of 16S rRNA gene was performed in a 20 μL volume containing 4 μL 5×FastPfu Buffer, 2 μL 2.5 mM dNTPs, 0.8 μL 5 μM forward primer, 0.8 μL 5 μM reverse primer, 0.4 μL 500 U FastPfu polymerase, 0.2 μL 20 mg mL^−1^ BSA, and 10 ng template DNA. The final volume was adjusted to 20 μL using ddH_2_O. The PCR products were extracted from a 2% agarose gel, purified using the AxyPrep DNA Gel Extraction Kit (Axygen Biosciences, Union City, CA, USA), and the Illumina MiSeq paired-end (PE300) sequencing was performed by Majorbio Biological Pharmaceutical Co., Ltd. (Shanghai, China). The raw amplicons reads were deposited in the NCBI Sequence Read Archive database under BioProject [PRJNA#878943].

### 2.4. Sequenced Data Processing

The raw reads were processed using the dada2 package in R [26]. The forward and reverse reads were demultiplexed by cutting off the barcode and primer sequences. Sequences with lengths greater than 200 bp and mean quality value ≥20 were retained. The chimeric sequences were removed by using the UCHIME algorithm [27]. Sequences were then clustered into amplicon sequence variants (ASVs). The taxonomic classification of the representative sequence for each ASV was performed using the Ribosomal Database Program classifier against the Silva 138 16S rRNA database [28]. Functional pathways were annotated by using the Tax4Fun2 package [29] in R, which is based on the Kyoto Encyclopedia of Genes and Genomes (KEGG) [30].

### 2.5. Statistical Analyses

The alpha diversity indexes (Chao1, Shannon) were calculated with the ASV table using the Vegan package (v 2.5.6) in R [31]. Differences in water chemical properties, bacterial community alpha diversity indexes, and phylum relative abundance among the rivers and reservoirs were tested using a one-way analysis of variance (ANOVA) of the linear fixed-effects model and Sidak test with an α of 0.05. The normality of residuals and homogeneity of variance assumptions were checked with Shapiro–Wilk and Levene’s tests, respectively, before conducting ANOVA. Natural base logarithmic transformations were applied to normalize the data if needed. A Venn diagram with shared and unique ASVs was applied to depict the similarities between soil bacterial communities of different groups. Principal coordinates analysis (PCoA) was performed based on the Bray–Curtis distances to visualize the composition of bacterial communities at the ASV level [32]. Permutation multivariate analysis of variance (PERMANOVA, Adonis function in the Vegan R package) with 999 random permutations were conducted to examine if water bacterial composition varies in different reaches and reservoirs of the Nandu River [33]. Canonical correlation analysis (CCA, “cca” function in the Vegan R package) was conducted to assess the correlation between bacterial communities (at the genus rank) and water properties [26]. In addition, Pearson correlation analysis was performed to examine the associations between water chemical properties and the diversity of water bacterial communities. All analyses were conducted using R Software (Version 4.1.3) [34].

## 3. Results

### 3.1. Properties of Water Samples

Water properties vary at different reaches of the Nandu River (Table 1). Water temperature was highest in the Songtao Reservoir and has no significant difference among different river reaches. Water DO was significantly higher in the Nandu River than the Songtao Reservoir. Water EC was highest at the lower reach and significantly higher than at the middle reach. Water turbidity was highest at the lower reach and significantly greater than at the upper reach and the reservoir. Water TN and NO_3_^−^ were significantly higher at the lower reach than at the upper reach and the reservoir. Water NO_2_^−^ was highest at the lower reach and significantly higher than at other reaches and the Songtao Reservoir. However, water pH, TP, COD_Mn_, NH_4_^+^, and PO_4_^3−^ were not significantly different between different reaches and the Songtao Reservoir.

### 3.2. The Bacterial Diversity of the Surface Water

**Figure 2:**
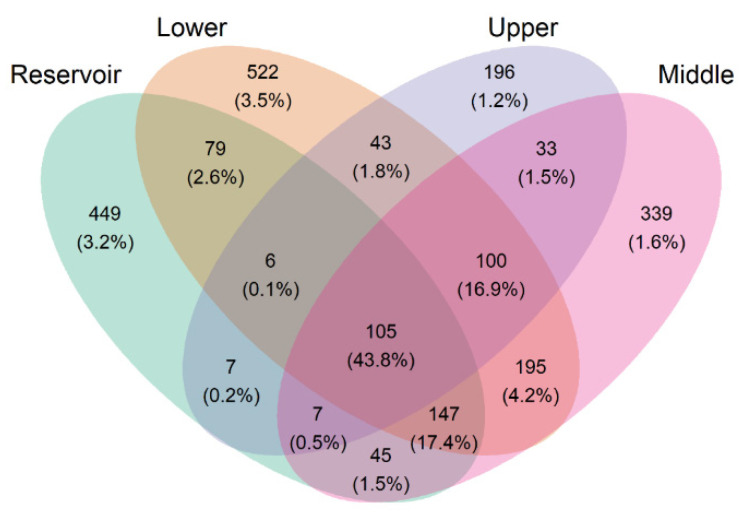
Venn diagram of ASVs and sequences of water sample groups. The numeral data are the ASV numbers and the percentage data are the ratio of the sequence numbers to the total sequence numbers.

In total, 389,264 high-quality sequences were obtained from the 14 surface water samples. The rarefaction analysis of ASVs (amplicon sequence variants) at >10,000 reads showed that the diversity of the 14 samples could be well represented (Figure S1). These high-quality sequences were clustered into 2273 ASVs (Figure 2). There were 43.8% shared sequences across the four different sample groups. In contrast, samples from the Songtao Reservoir and the lower reach of the Nandu River had a relatively higher percentage of unique sequences, with values of 3.2% and 3.5%, respectively (Figure 2).

**Figure 3:**
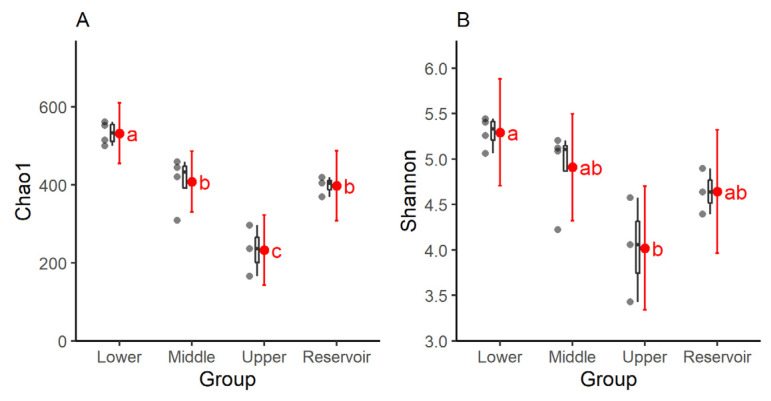
Bacterial (**A**) Chao1 and (**B**) Shannon values of the lower, middle, and upper reaches of the Nandu River and the Songtao Reservoir. Means not sharing any lowercase letter indicating significant differences among different groups.

The Chao1 value (richness) of the water samples drawn from the upper reach was the smallest and significantly lower than at other reaches and the Songtao Reservoir (Figure 3A). The Shannon value (diversity) of the water samples drawn from the upper reach was the smallest and significantly lower than that sampled from the lower reach, but has no significant differences with samples drawn from the middle reach and the Songtao Reservoir (Figure 3B).

### 3.3. Bacterial Community Structure and Composition in the Water Samples

**Figure 4:**
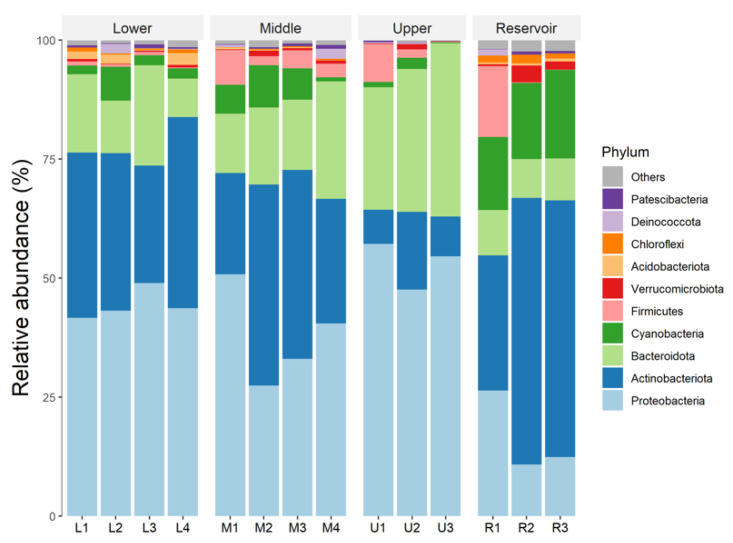
The relative abundance of dominant bacterial phyla (top 10) in the water samples.

At the phylum rank, on average, the dominant bacterial phyla were Proteobacteria (38.41%, relative abundance, same below), Actinobacteriota (30.88%), Bacteroidota (17.39%), and Cyanobacteria (6.38%), with the relative abundance of the top 10 bacterial phyla accounting for 98.88% of the bacterial sequences across all 14 water samples (Figure 4). However, Proteobacteria and Actinobacteriota were the most dominant phylum in the river and reservoir, respectively (Figure 4). For the top 10 phyla, their relative abundance was significantly different in reservoir samples and some reaches of river samples, such as Proteobacteria, Actinobacteriota, Bacteroidota, Cyanobacteria, and Chloroflexi (Table S1). However, the relative abundance of Firmicutes, Acidobacteriota, Patescibacteria, and Deinococcota had no significant difference in the reservoir and the river (Table S1).

**Figure 5:**
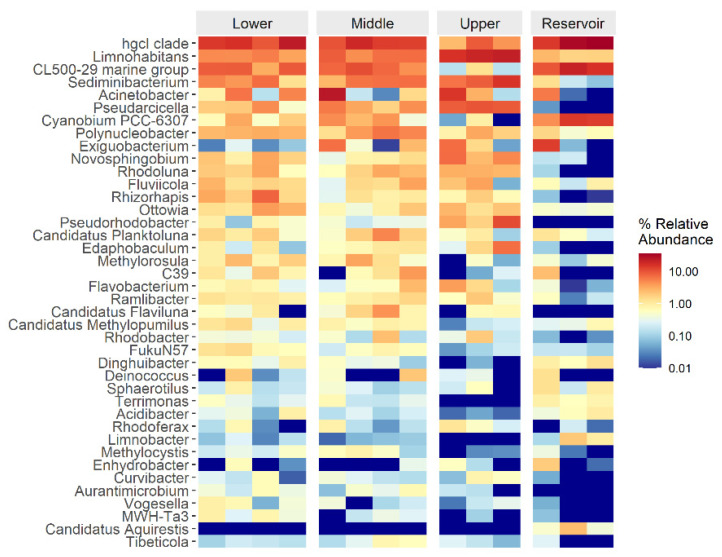
Top 40 taxa at genus level of the 14 water samples.

A noticeable difference was observed at the genus rank between river and reservoir samples (Figure 5). The hgcI clade, Limnohabitans, and CL500-29 marine group were the dominant genus in both river and reservoir samples (Figure 5). However, the relative abundance of Novosphingobium, Rhodoluna, Rhizorhapis, and Pseudorhodobacter was found to be high in river samples but not in reservoir samples (Figure 5).

**Figure 6:**
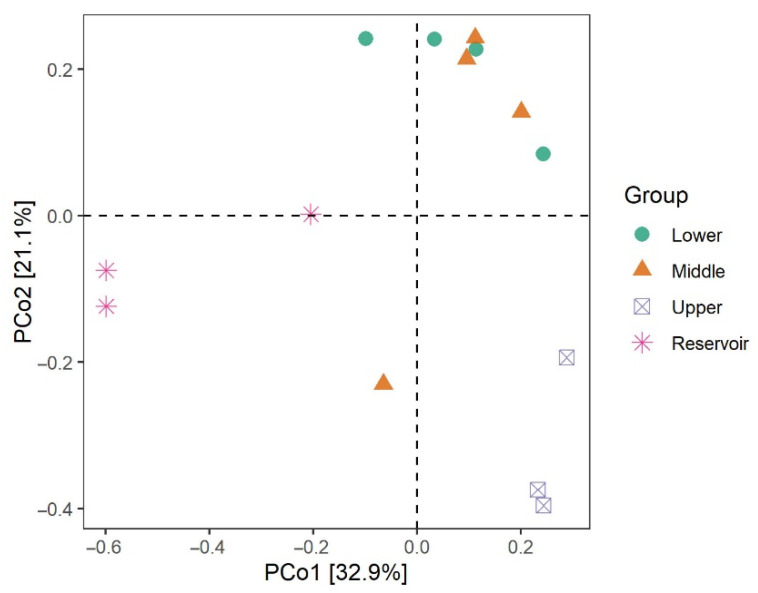
Principal coordinate analysis (PCoA) plots of bacterial communities in the water.

The PCoA plot and PerMANOVA result (F = 3.27, *p* < 0.001) clearly show a separation between the reservoir and river samples on the first and second axis, which together accounted for 54.0% of the variation (Figure 6).

### 3.4. Correlation between Bacterial Communities and Environmental Factors

**Figure 7:**
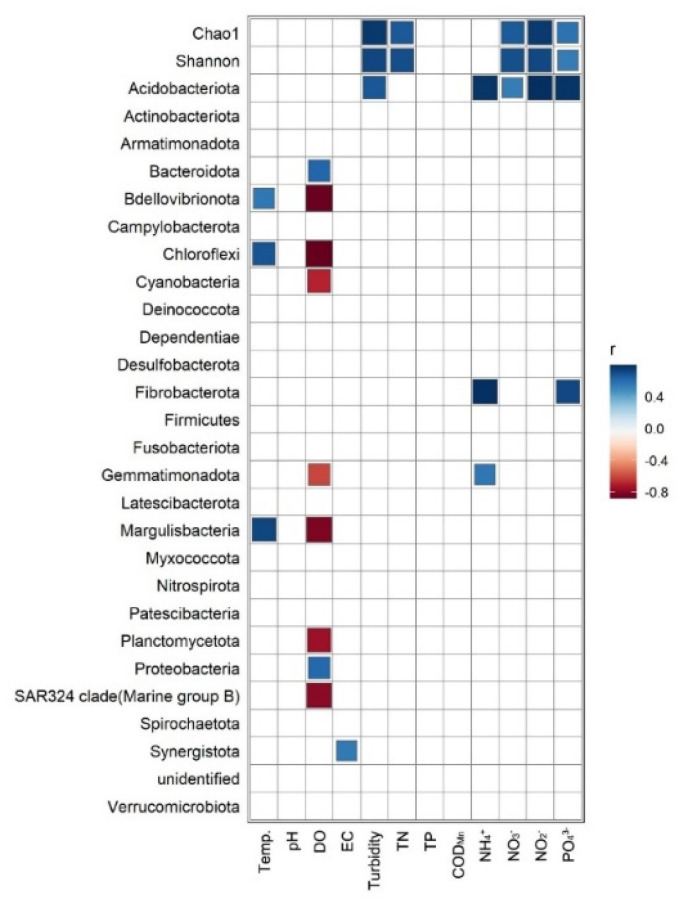
Correlation between environmental variables and the relative abundance of bacterial phyla and alpha diversity indexes.

**Figure 8:**
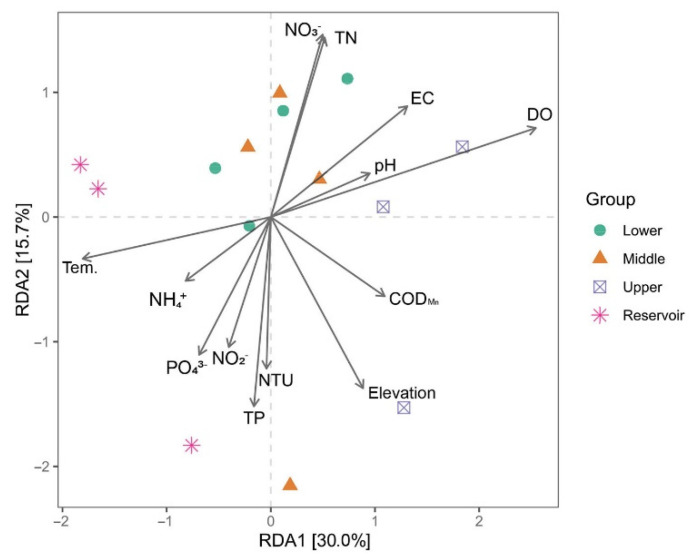
Canonical correspondence analysis (CCA) plots of bacterial communities in the water.

The correlation between environmental variables and dominant phyla indicated that DO was positively correlated with the relative abundance of Proteobacteria and Bacteroidota (Figure 7). DO also negatively correlated with the relative abundance of Bdellovibrionota, Chloroflexi, Cyanobacteria, Gemmatimonadota, Margulisbacteria, Planctomycetota, and SAR324 clade (Marine group B) (Figure 7). Likewise, the NH_4_^+^ and PO_4_^3−^ concentrations were negatively correlated with the relative abundance of Fibrobacterota (Figure 7). The Pearson correlation between environmental variables and alpha diversity indexes showed that NTU, TN, NO_3_^−^, NO_2_^−^, and PO_4_^3−^ were positively correlated with both the Chao1 and Shannon indexes (Figure 7). CCA of the bacterial genus was used to identify their relationship with environmental variables, with the first two CCA axes explaining 45.7% of the total variance in the bacterial composition (at the genus rank). The Mantel test was used to check whether there were significant correlations between environmental variables and the bacterial communities’ distance matrix, DO (*p* = 0.018), TN (*p* = 0.050), and NO_3_^−^ (*p* = 0.040) identified as significant (Figure 8).

### 3.5. Bacterial Functional Genes

**Figure 9:**
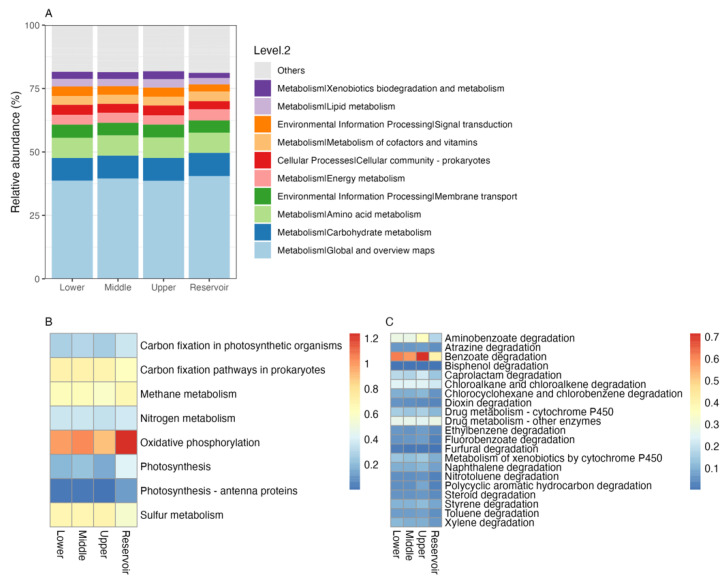
Function prediction obtained by Tax4Fun2. (**A**) Functional gene pathways at KEGG level2; (**B**) relative abundance of functions under the energy metabolism category; (**C**) relative abundance of functions under the xenobiotics’ biodegradation and metabolism category.

Tax4Fun2 analysis indicated that the major functional gene groups were related to metabolism (76.08~78.35%), environmental information processing (8.17~8.97%), cellular process (4.47~5.81%), genetic information processing (4.09~5.58%), human diseases (2.59~3.37%), and organismal systems (1.37~1.49%) (Figure 9A). The relative abundance of most predicted functions was significantly different between the Nandu River and Songtao Reservoir. For example, the relative abundance of genetic information processing was significantly higher in the Songtao Reservoir than in the Nandu River, while the relative abundance of human diseases was significantly lower in the Songtao Reservoir than in the Nandu River (Table S2). Under the metabolism category, genes related to global and overview maps, carbohydrate metabolism, amino acid metabolism, energy metabolism, and xenobiotics biodegradation and metabolism had high abundance in all groups. We further investigated the energy metabolism and the xenobiotics biodegradation categories at KEGG level 3, with the results shown using heatmaps (Figure 9B,C). The oxidative phosphorylation, carbon fixation pathways in prokaryotes, methane metabolism, and sulfur metabolism pathways had higher relative abundance than others under the energy metabolism category. The benzoate degradation and aminobenzoate degradation pathways had higher relative abundance than others under the xenobiotics biodegradation category. The upper reach had the highest relative abundance of the benzoate degradation pathway.

## 4. Discussion

Our results indicated significant differences in the water microbial diversity and its community composition at different reaches of the Nandu River and the Songtao Reservoir. A change in surface water can change microbial communities, leading to changes in water quality and contamination by water-borne pathogens [35]. The upper reach had the lowest bacterial richness and diversity, indicated by the Chao1 and Shannon values, probably due to the water flowing faster and fewer anthropogenic activities delivering fewer organic contaminants into the river. This finding is consistent with Godoy, who reported that reservoirs with less anthropogenic influences had lower Shannon values than rivers in Brazil [18].

Our study found that the four groups (upper, middle, and lower reaches of the Nandu River and the Songtao Reservoir) shared only 105 core ASVs but occupied 43.8% of all sequences (Figure 2). As a result, these core species (ASVs) may be well adapted to tropical surface waters. The dominant bacterial phyla in river samples were Proteobacteria (Figure 4). This is consistent with other studies demonstrating Proteobacteria as a rich bacterial phylum observed in surface water by analysis of the hypervariable regions of the 16S rRNA gene [36,37]. However, we found that Actinobacteriota was the dominant bacterial phylum in reservoir samples. This agrees with previous studies, which found that Actinobacteriota was dominant in lakes [36,38,39]. Actinobacteriota can enter the drinking water reservoir through rainfall or surface runoff, as reported by Zhang et al. [40]. Furthermore, the hgcI clade (Actinobacteriota) had the highest relative abundance in both river and reservoir samples in our study, possibly because it is a common and abundant bacterial clade that is tolerant of a wide range of water conditions [41,42]. On the other hand, the abundance of the hgcI clade communities, has been shown to be positively correlated with solar ultraviolet (UV) radiation [43]. The reservoir locates at a higher elevation and has a wide range of open water surfaces that receive more UV radiation, which might explain why the relative abundance of this genus has higher relative abundance in the reservoir than in the river. Furthermore, a higher relative abundance of Cyanobacteria was found in reservoir samples than in river samples (Figure 4). This could be attributed to reservoirs usually having a stable water environment compared to rivers and favoring eutrophication conditions [44]. However, in our study, both the Nandu River and Songtao Reservoir do not have eutrophication problems, indicating that the nitrogen and phosphorus concentrations were still at a relatively lower level [45] (Table 1).

Water turbidity and nutrients were positively correlated with bacterial Chao1 and Shannon diversity indexes in this study, which confirmed turbidity, or water resident time, could significantly influence microbial diversity, as water resident time could be measured by turbidity [46]. Furthermore, nutrients were also positively correlated with Chao1 and Shannon diversity indexes, indicating the importance of nutrients as the energy source for microbes [47]. Except for nutrients, DO was also important for microbes in the water system, as indicated by the RDA results. These microbes can use DO to decompose organic material and are essential for nutrient recycling in water [48]. However, unlike many other studies that show pH is an essential factor in determining microbial diversity, probably due to the pH only ranging from 7.25~7.50 in our study, it did not cause significant changes in the bacteria community.

Tax4Fun2 is a unique tool that accurately predicts functional profiles of prokaryotic communities from 16S rRNA gene sequences [49]. The relative abundances of functional profiles were not significantly different among different reaches of the Nandu River and the Songtao Reservoir (Figure 9), probably due to the water quality being in good condition. It has been reported that significant differences in functional profiles can be found at sites with different levels of pollution in the same river [50]. However, the appearance of xenobiotics biodegradation and metabolism-related genes indicates the presence of pollutants in the tropical river and reservoir ecosystem. On the other hand, a higher relative abundance of benzoate and aminobenzoate degradation pathways was found in river samples, indicating that the Nandu River may contain more pollutants than the Songtao Reservoir. Some natural or industrial pollutants may enter the river at its upper reaches, and their concentration can be diluted in the reservoir. Thus, more efforts should be made to protect the drinking water resources, especially at the upper reach of the Nandu River, which is one of the sources of the Songtao Reservoir. Furthermore, it provides an opportunity to exploit microbial resources capable of bioremediating waste materials [51].

## 5. Conclusions

In conclusion, water properties show significant differences between different reaches of the Nandu River and the Songtao Reservoir, especially water temperature, DO, and nutrients. The highest species’ richness and diversity were found in the lower reach of the Nandu River, based on the Chao1 index and Shannon index. The beta diversity analysis indicated the divergence in bacterial communities in the Nandu River and Songtao Reservoir. At the genus rank, the bacterial composition was very similar in the lower and middle reaches of the Nandu River, while the upper reach of the Nandu River was similar to the Songtao Reservoir. Proteobacteria and Actinobacteriota were the most abundant phyla in the Nandu River and Songtao Reservoir, respectively. The hgcI clade showed high relative abundance in all samples. Functional profiles predicted by Tax4Fun2 showed similar patterns in water samples, while the benzoate and aminobenzoate degradation pathways had higher relative abundance in the upper reach of the Nandu River. Our study showed baseline information on the changes in bacterial communities of the water bodies connected to the Nandu River.

## Figures and Tables

**Table 1:** Properties of water samples drawn from the Nandu River. Values are means ± SE, means not sharing any lowercase letter indicating significant differences among different groups.

Properties	Reservoir	Upper	Middle	Lower
Temperature (°C)	25.87 ± 1.26 a	23.57 ± 0.60 ab	23.33 ± 1.19 b	25.00 ± 0.91 ab
pH	7.20 ± 0.30 a	7.50 ± 0.48 a	7.29 ± 0.15 a	7.35 ± 0.29 a
DO (mg L^−1^)	7.05 ± 0.76 b	8.53 ± 0.16 a	8.47 ± 0.10 a	8.08 ± 0.28 a
EC (uS cm^−1^)	81.27 ± 16.31 ab	96.37 ± 11.63 ab	78.30 ± 19.68 b	117.28 ± 11.90 a
Turbidity	4.36 ± 2.00 b	4.24 ± 1.19 b	11.33 ± 2.95 ab	17.13 ± 4.85 a
TN (mg L^−1^)	0.86 ± 0.20 c	1.84 ± 1.06 bc	4.75 ± 2.00 ab	6.28 ± 1.68 a
TP (mg L^−1^)	0.10 ± 0.05 a	0.13 ± 0.06 a	0.15 ± 0.06 a	0.17 ± 0.07 a
COD_Mn_ (mg L^−1^)	0.75 ± 0.18 a	4.69 ± 5.91 a	1.28 ± 0.91 a	2.88 ± 3.68 a
NH_4_^+^ (mg L^−1^)	0.05 ± 0.03 a	0.04 ± 0.04 a	0.06 ± 0.03 a	0.29 ± 0.34 a
NO_3_^−^ (mg L^−1^)	0.59 ± 0.49 c	1.56 ± 1.31 bc	4.43 ± 2.04 ab	6.07 ± 1.52 a
NO_2_^−^ (mg L^−1^)	0.02 ± 0.03 b	0.01 ± 0.01 b	0.03 ± 0.02 b	0.09 ± 0.02 a
PO_4_^3−^ (mg L^−1^)	0.03 ± 0.01 a	0.02 ± 0.01 a	0.08 ± 0.05 a	0.14 ± 0.09 a

## Data Availability

The raw data in this study were deposited in the NCBI Sequence Read Archive database with accession number PRJNA878943.

## Supplementary Materials

The following supporting information can be downloaded at: https://www.mdpi.com/article/10.3390/ijerph20010382/s1, Figure S1: Rarefaction curves for samples in the Nandu River and Songtao Reservoir; Table S1: Relative abundance of the dominant phyla of water samples drawn from different reaches of the Nandu River and the Songtao Reservoir; Table S2: Relative abundance of the functional genes of water samples drawn from different reaches of the Nandu River and the Songtao Reservoir.
