# Supplementary material for: Bacterial Community Composition and Function of Tropical River Ecosystem along the Nandu River on Hainan Island, China
Source: Int J Environ Res Public Health. 2022 Dec 26;20(1):382. doi: 10.3390/ijerph20010382 (PMC9819888; doi:10.3390/ijerph20010382)
Supplement: Supplementary file 1 [file ijerph-20-00382-s001.zip › ijerph-1936624-supplementary.pdf]

Article

Bacterial Community Composition and Function of Tropical River Ecosystem along the Nandu River on Hainan Island, China

Jinbiao Li [1,2,3], Yangni Zhai [1,3], Guojian Ge [1,3], Yang Xu [1,3], Can Wang [1,3], Anyong Hu [1,3], Yujie Han [1,3], Nan Shan [2,3], Bo Liu [1,3,*], Jinlin Chen [4] and Wenlin Wang [2,3,*]

[1] School of Geographic Science, Nantong University, Nantong 226019, China
[2] Nanjing Institute of Environmental Sciences, Ministry of Environmental Protection, Nanjing 210042, China
[3] State Environmental Protection Scientific Observation and Research Station for Ecological Environment of Lake Hulun Wetland, Hulun Buir 021000, China
[4] Co-Innovation Center for Sustainable Forestry in Southern China, Nanjing Forestry University, Nanjing 210037, China
* Correspondence: lb@ntu.edu.cn (B.L.); wangwenlin_jjl@126.com (W.W.)

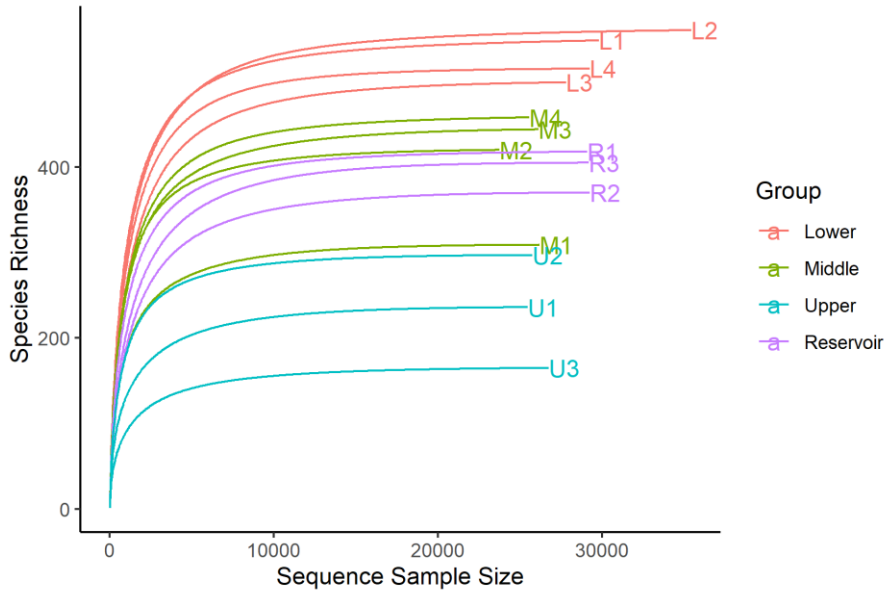

Figure S1. Rarefaction curves for samples in the Nandu River and Songtao Reservoir.

Table S1. Relative abundance of the dominant phyla of water samples drawn from different reaches of the Nandu River and the Songtao Reservoir.

Phylum	Reservoir	Upper	Middle	Lower
Proteobacteria	16.52 ± 4.21 b	53.10 ± 4.21 a	37.90 ± 3.65 ab	44.33 ± 3.65 a
Actinobacteriota	46.09 ± 5.65 a	10.62 ± 5.65 b	32.37 ± 4.90 ab	33.18 ± 4.90 ab
Bacteroidota	8.85 ± 2.85 b	30.72 ± 2.85 a	17.03 ± 2.47 b	14.16 ± 2.47 b
Cyanobacteria	16.65 ± 1.43 a	1.24 ± 1.43 b	5.60 ± 1.24 b	3.31 ± 1.24 b
Firmicutes	5.01 ± 2.56 a	3.29 ± 2.56 a	3.96 ± 2.21 a	0.56 ± 2.21 a
Verrucomicrobiota	1.88 ± 0.45 a	0.41 ± 0.45 a	0.60 ± 0.39 a	0.38 ± 0.39 a
Acidobacteriota	0.51 ± 0.31 ab	0.02 ± 0.31 b	0.22 ± 0.27 b	1.52 ± 0.27 a
Chloroflexi	1.44 ± 0.15 a	0.02 ± 0.15 b	0.20 ± 0.13 b	0.54 ± 0.13 b
Patescibacteria	0.40 ± 0.16 a	0.12 ± 0.16 a	0.46 ± 0.14 a	0.44 ± 0.14 a
Deinococcota	0.44 ± 0.49 a	0.20 ± 0.49 a	0.65 ± 0.42 a	0.55 ± 0.42 a

Values are means ± SE. Means not sharing any lowercase letter indicate significant differences among groups ($p < 0.05$).

Table S2. Relative abundance of the functional genes of water samples drawn from different reaches of the Nandu River and the Songtao Reservoir.

Function	Reservoir	Upper	Middle	Lower
Metabolism	78.35 ± 0.37 a	76.56 ± 0.37 b	77.20 ± 0.32 ab	76.08 ± 0.32 b
Environmental Information Processing	8.17 ± 0.25 b	8.68 ± 0.25 ab	8.35 ± 0.22 ab	8.97 ± 0.22 a
Cellular Processes	4.47 ± 0.22 b	5.80 ± 0.22 a	5.12 ± 0.19 ab	5.81 ± 0.19 a
Genetic Information Processing	5.58 ± 0.19 a	4.09 ± 0.19 b	4.77 ± 0.17 b	4.48 ± 0.17 b
Human Diseases	2.59 ± 0.06 c	3.37 ± 0.06 a	3.11 ± 0.06 b	3.24 ± 0.06 ab
Organismal Systems	1.37 ± 0.03 b	1.49 ± 0.03 a	1.44 ± 0.02 ab	1.41 ± 0.02 ab

Values are means ± SE. Means not sharing any lowercase letter indicate significant differences among groups ($p < 0.05$).
